# An Unusual Case of Metachronous Tumors of Prostate and Parotid Gland: A Diagnostic Dilemma

**DOI:** 10.7759/cureus.48477

**Published:** 2023-11-07

**Authors:** Yanko G Yankov, Lyuben Stoev, Katerina Stanislavova, Simeon Dimanov

**Affiliations:** 1 Clinic of Maxillofacial Surgery, University Hospital St. Marina, Varna, BGR; 2 Department of General and Operative Surgery, Medical University "Prof. Dr. Paraskev Stoyanov", Varna, BGR; 3 Department of General and Clinical Pathology, Forensic Medicine, and Deontology, Medical University "Prof. Dr. Paraskev Stoyanov", Varna, BGR; 4 Department of Oral Surgery, Medical University "Prof. Dr. Paraskev Stoyanov", Varna, BGR

**Keywords:** pet/ct in salivary gland tumors, fine needle aspiration biopsy of the parotid gland, ductal breast carcinoma, prostate gland, oral pathology, maxillofacial surgery, head and neck surgery, prostate adenocarcinoma, parotid gland, salivary gland tumors

## Abstract

If diagnosed early, prostate cancer (PCa) has a good prognosis and an excellent five-year survival rate. However, this favorable behavior can be drastically worsened by the presence of another synchronous or metachronous higher-grade malignancy. In the current case report, we present and analyze a 76-year-old patient who underwent radical prostatectomy because of prostate gland adenocarcinoma, diagnosed on needle biopsy. The low Gleason score and the early stage of the PCa are in significant contrast with the widespread metastatic disease that is observed during the follow-up. Additional clinical examination, imaging, and histological evaluation reveal a high-grade salivary duct carcinoma, a metachronous primary in the left parotid gland. The presence of these two malignancies raises a series of diagnostic difficulties faced by medical professionals, in part because of the tendency of prostate gland and salivary gland tumors to show some overlapping features regarding their biological behavior and immunohistochemical aspects.

## Introduction

Salivary gland tumors (SGTs) comprise a rare group of neoplasms that pose a diagnostic challenge due to their rarity of occurrence and diversity of types. The majority of SGTs tend to be benign. In contrast, malignant tumors originating in the salivary glands are relatively rare, accounting for less than 5% of all head and neck neoplasms. Salivary duct carcinoma (SDC) is a rare tumor constituting a mere 1-3% of the total cases of malignant SGTs. Its etiology is so far unknown. Potential contributing factors encompass cigarette smoking, employment in rubber manufacturing, genetic predisposition, and viral infections among others. Radiation exposure has been clearly established as a risk factor for parotid malignancies [1].

SDC is a rare type of carcinoma but is thought to be a distinct malignancy of the major salivary glands because of its highly aggressive behavior and resemblance to high-grade ductal carcinoma of the breast. SDC exhibits rapid growth, pain, facial palsy, and nodal metastasis in a significant proportion of patients at presentation [2]. The prognosis is poor with a five-year overall survival of 43% and a mean five-year recurrence-free survival of 34% [3]. One of the primary reasons for treatment failure in SDC is the development of distant metastases, which significantly impacts the prognosis, typically resulting in a median survival of 4.3-7.3 months [4]. The course of the disease is aggravated in the presence of a synchronous malignant process, as in the patient we describe. In this case, the presence of two oncological diseases (SDC of the left parotid gland and prostate adenocarcinoma) led to a series of puzzles to be solved, the answer to which will be found in the present case report.

## Case presentation

**Figure 1:**
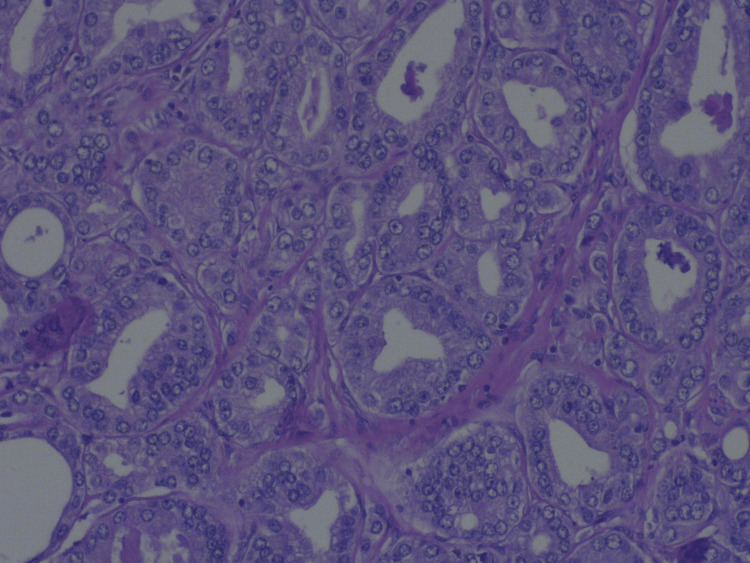
Photomicrograph revealing prostate gland adenocarcinoma with predominant pattern of glands with "back to back" arrangement and lined by cells with enlarged vesicular nuclei and nucleolar prominence, H&E x40

A 76-year-old male patient presented to an oral and maxillofacial surgeon in July 2022 with a progressively enlarging lump on the left side of his neck, creating a sense of discomfort but without any major clinical symptoms. The patient had hypertension, ischemic heart disease (IHD), and hyperlipidemia as accompanying diseases, which are stable on the background of regular intake of oral medications (amiodarone, lisinopril, torasemide, bisoprolol, and rosuvastatin). He did not report any family history of neoplastic diseases or food and drug allergies. As part of his past history, it was revealed that in June 2021, because of a diagnosed prostate gland adenocarcinoma (Figure 1), the patient underwent radical prostatectomy by robot-assisted surgery (Da Vinci Xi, Intuitive Surgical, Inc., Sunnyvale, California, United States) in an urology clinic.

Preceding the surgical intervention, on the occasion of elevated prostate-specific antigen (PSA) values ​​of 10.11 ng/ml (normal range 1-1.5 ng/ml), an MRI-ultrasound fusion biopsy of the prostate gland was performed with a histopathological result of adenocarcinoma with Gleason score 3+4=7, International Society of Urologic Pathologists (ISUP) Grade group 2. A whole-body bone scintigraphy with 20mCi 99mTc multidrug-resistant tuberculosis (TB) test was performed in May 2021 and no abnormalities of the distribution of the osteotropic radiopharmaceutical were found. A whole-body prostate-specific membrane antigen (PSMA) positron emission tomography (PET)/CT in April 2022 showed radiopharmaceutical accumulation in the following areas: in both lungs without increased PSMA activity, in the right half of the L2 vertebra with increased PSMA activity, and under the anterior abdominal wall on the right with increased PSMA-activity, with no increased accumulation in the bed of the removed prostate gland and in the head and neck region. PSA level of <0.01 ng/ml was then measured. On the basis of all the studies carried out after discussion by a medical board, it was concluded that the increased activity under the anterior abdominal wall is most likely due to granuloma, but it may also be the result of secondary involvement from another synchronous process; the increased activity in the body of vertebra L2 is most likely an osteophyte and of osteodegenerative origin; that there is no recurrence of carcinoma of the prostate gland and there is no regional malignant lymphadenopathy (miT0, miN0).

The patient was scheduled for a follow-up CT with intravenous contrast material in June 2022, which showed enlarged lesions in both lungs and under the anterior abdominal wall on the right compared to previous imaging, with a new one in the right liver lobe (VII segment). The patient was prescribed chemotherapy (four courses of docetaxel every 21 days).

**Figure 2:**
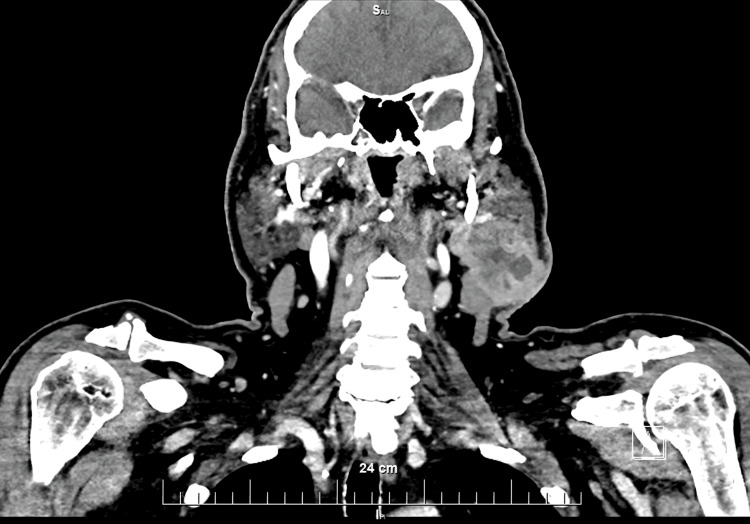
Axial CT scan of the head and neck showing the tumor mass in the left parotid gland. The tumor mass involves the two lobes of the left parotid gland and the facial nerve between them. The Ill-defined margins demonstrated by the lesion are strongly suggestive for malignancy.

**Figure 3:**
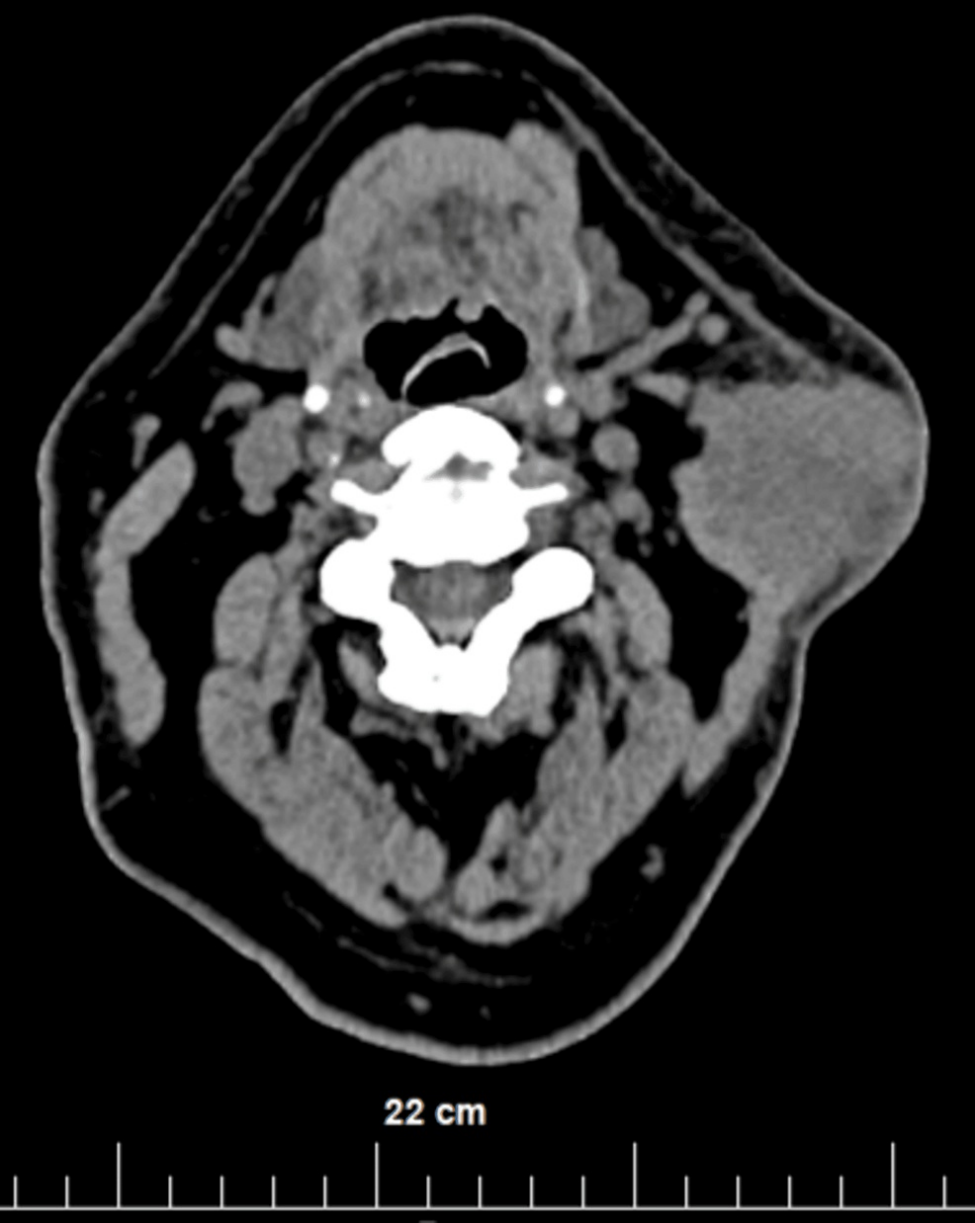
Coronal CT scan of the head and neck showing the tumor mass in the left parotid gland. The tumor mass involves the two lobes of the left parotid gland and the facial nerve between them. The Ill-defined margins demonstrated by the lesion are strongly suggestive for malignancy.

In July 2022, the physical examination by a maxillofacial surgeon revealed the presence of a painless, hard-elastic lump in the left parotideomasseteric region, about 5.0 cm in diameter, and dark purple color of the skin above it, the latter without evidence of ulceration. A CT with venous contrast material of the head and neck was ordered, which showed the presence of a primary tumor process of the left parotid gland of an infiltrative nature, without evidence of secondary involvement of lymph nodes in the neck region (Figure 2 and Figure 3).

**Figure 4:**
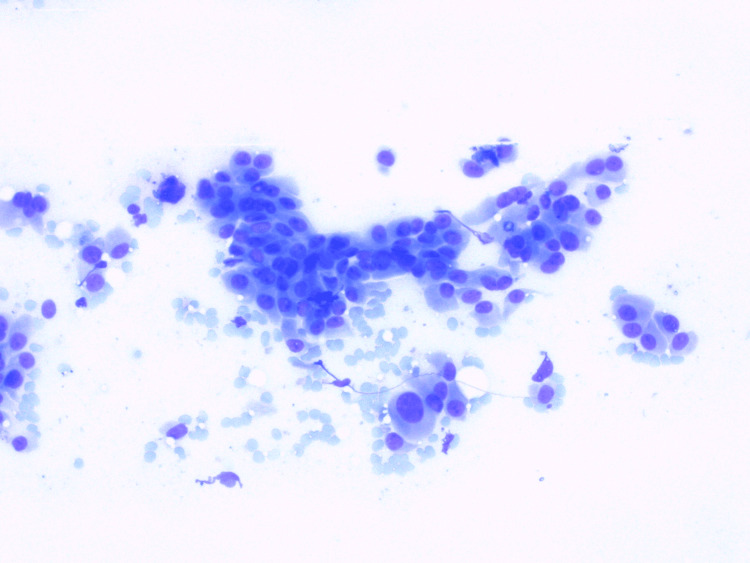
FNA cytological specimen shows numerous clusters of cells with ample cytoplasm, anisocariosis, enlarged nuclei with prominent nucleoli, focal glandular arrangement, Giemsa x20 FNA: fine-needle aspiration

The patient underwent a fine-needle aspiration biopsy of the lesion. The pathological report revealed a neoplastic category suggestive for a high-grade malignancy (Figure 4).

**Figure 5:**
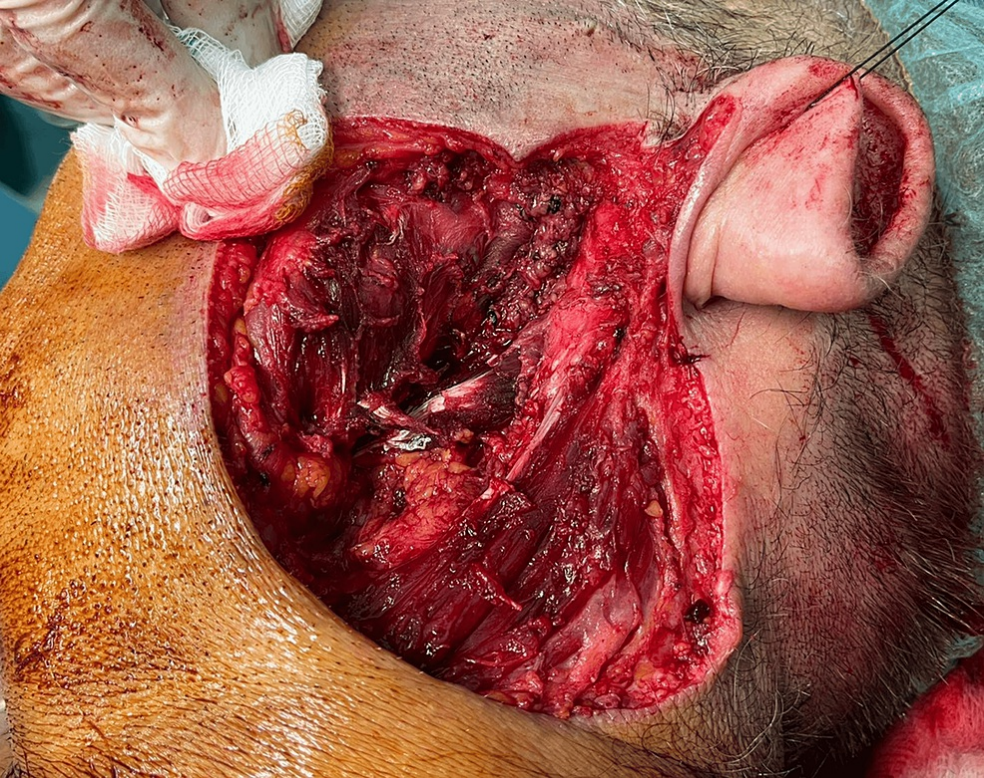
Intraoperative image showing the bed of the completely extirpated parotid gland along with the excised facial nerve, the attachments of the adjacent muscles, and the overlying skin

The patient was hospitalized after the current presentation, in August 2022, and a radical parotidectomy was performed. This involved the removal of the left parotid gland along with all the visibly affected surrounding soft tissues, including the overlying skin, subcutaneous tissue, fascia, and the engaged portions of the following muscles: the lateral part of the posterior belly of the digastric muscle, the anterior-superior part of the sternocleidomastoid muscle, and the posterior part of the masseter muscle. A selective neck dissection with removal of the II-level ipsilateral lymph nodes was performed. Excision of the overlying tissues was performed because of their gross infiltration by the process; the facial nerve was also resected because its trunk and the proximal parts of its five branches showed evidence of tumor involvement (Figure 5).

**Figure 6:**
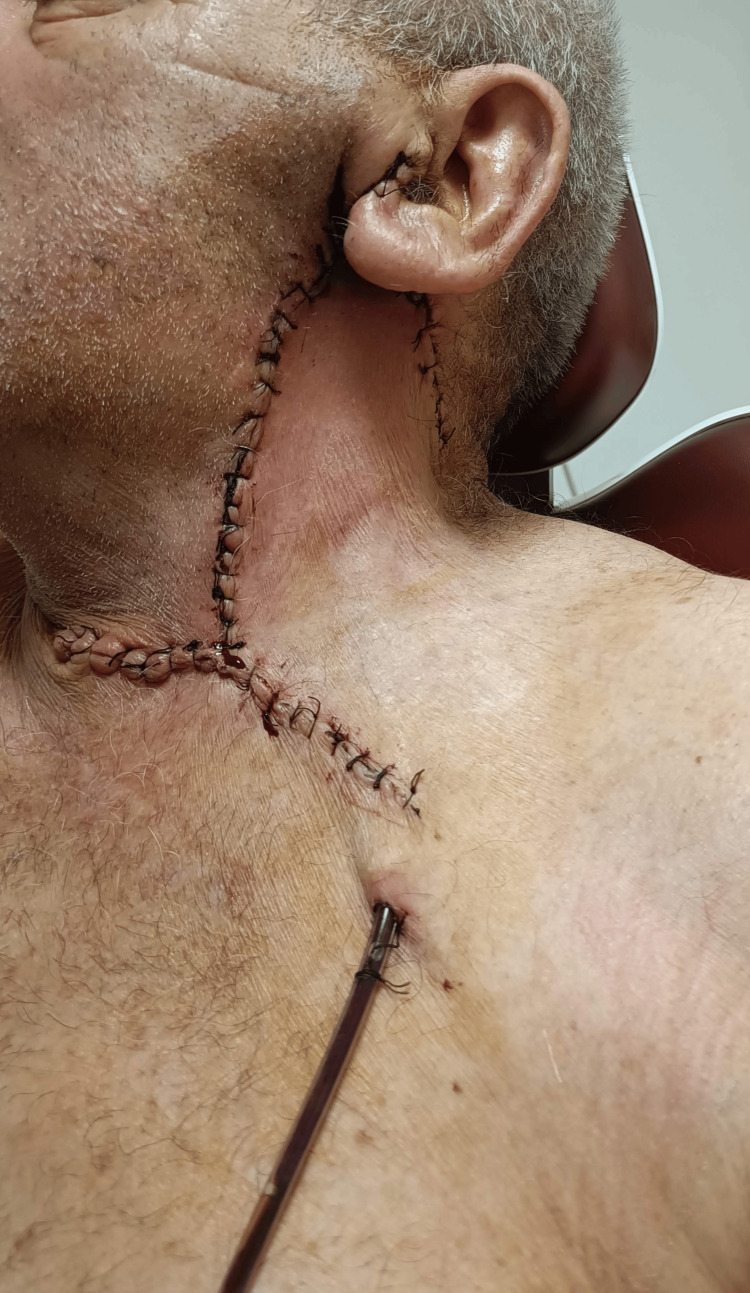
The placed vacuum drain and relaxed surgical wound are visualized along with plastic repair on the third postoperative day

After thorough hemostasis, a vacuum Redon drain was placed and the wound was sutured with resorbable polyfilament for muscles, fascia, and subcutaneous tissue and with non-resorbable polyfilament for skin. The postoperative period was uneventful and with surgical antibiotic prophylaxis with cefazoline (2 g three times intravenously) for three days. On the third postoperative day, the drainage, which contained 40 mL of blood and transudate, was removed and the patient was discharged without home therapy (Figure 6).

**Figure 7:**
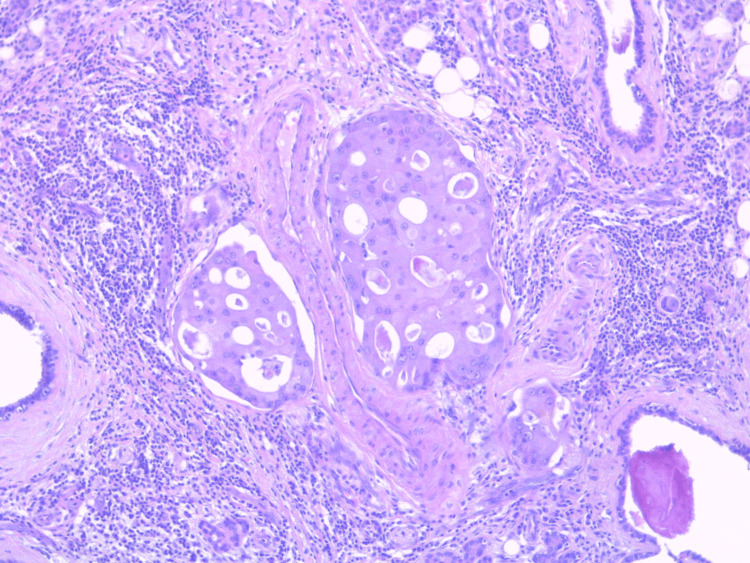
Vascular emboli of a tumor with cribriform growth pattern. Cytoplasm is dense and eosinophilic; there is phenotypical similarity to ductal carcinoma of breast, H&E x20

**Figure 8:**
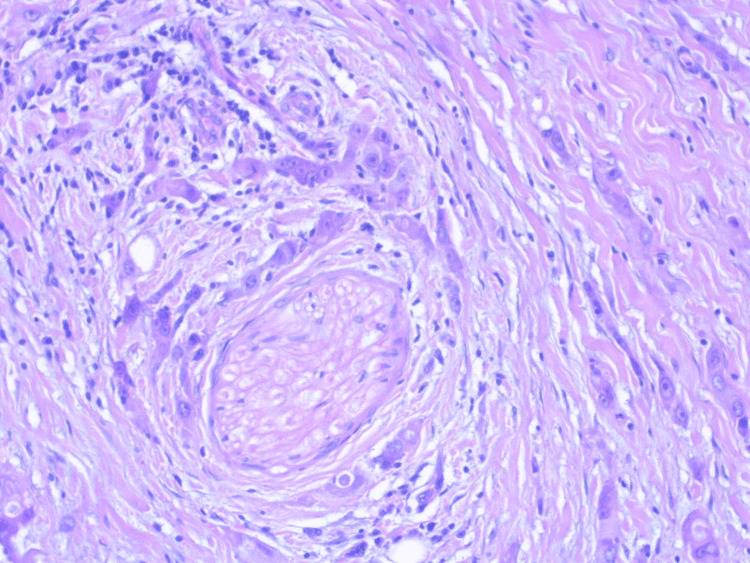
Infiltrative tumor cells with markedly increased nuclei and nucleolar prominence showing trabecular formation in a focus of perineural invasion, H&E x40

The sutures were removed on the 10th postoperative day. The histological result was “high-grade SDC of the parotid gland” and revealed multiple tumor emboli, no involvement of the surgical margins, and no metastasis in the removed lymph nodes (Figure 7 and Figure 8).

Postoperatively, a whole body PET/CT with 18F-fluorodeoxyglucose (FDG) 4.5mCi was performed, showing no local recurrence and no secondary involvement of cervical lymph nodes, an increased radiopharmaceutical accumulation was visualized bilaterally in the lungs, under the right anterior abdominal wall, in the VII segment of the liver and in the right halves of the L1 and L2 vertebrae, all significantly increased in the area compared to the previous imaging. The patient is administered a new course of chemotherapy.

A new follow-up CT with venous contrast material of the affected areas in the abdomen and chest performed in April 2023 revealed progression of the metastatic disease, manifested by an increase in the size of all previously described metastatic lesions, infiltration of the anterior abdominal wall and colon, and infiltration of the right psoas muscle from the metastasis in L1 and L2 vertebrae.

## Discussion

SDC was first described by Kleinsasser and colleagues in 1968 as a malignant tumor of salivary gland origin with a striking resemblance to ductal carcinoma of breast. It was recognized as a separate entity by the World Health Organization (WHO) in 1991 [5]. SDC usually presents as an aggressive neoplasm, forming a bulky mass with frequent extraglandular extension and regional spread [6]. Most SDCs originate from the major salivary glands, the parotid gland being the most common site (84.7%); some tumors in the minor salivary glands, sinonasal tract, and lacrimal gland have also been reported [7,8].

SDCs are either de novo lesions or evolve from pre-existing pleomorphic adenoma [5]. The cells of SDC are characterized by abundant eosinophilic granular cytoplasm; nuclei usually show substantial polymorphism and nucleolar prominence. Foci with comedonecrosis are very common. Several histological subtypes are currently recognized: basal-like, mucin-rich, micropapillary, sarcomatoid, and rhabdoid. They rarely pose diagnostic problems as they are always associated with conventional areas [6].

Although not universally utilized, Nakaguro et al. suggested a useful risk stratification model, based on several histological features; prominent nuclear pleomorphism, ≥ 30 mitoses/10 high-power field (HPF), vascular invasion, ≥ 5 tumor budding, and ≥ 5 poorly differentiated clusters were strong prognostic predictors of a poor overall survival (OS) or progression-free survival (PFS) [9].

The most common sites of metastasis are lung (65%), bone (13%), liver (4%), and the CNS (4%). Other sites include nonregional lymph nodes, pleura, pericardium, orbit, skin, adrenal, and thyroid glands [4,6]. Due to the high incidence of loco-regional recurrence and distant metastasis resulting in very low survival outcomes, an aggressive approach should be the mainstay of treatment [10].

The therapeutic approach in SDC is wide surgical resection along with lymph node dissection followed by adjuvant radiation therapy. The role of adjuvant chemotherapy and targeted therapies has limited benefit to date. OS of the metastatic disease is poor, and 60-80% of patients with advanced-stage die within three years [11].

We presented a case report of a 76-year-old male patient with organ-confined prostate adenocarcinoma and metachronous SDC of the parotid gland with metastatic spread in the lungs, liver, bones, and CNS. High-grade adenocarcinomas metastatic to a major salivary gland are routinely considered in the differential diagnosis of SDC [11].

Metastasis from the prostatic primary in the current case was considered highly unlikely because of the very early pathological stage (pT2) and low-risk histological grade group (Gleason score 3+4, ISUP Grade group 2) [12]. The lack of extraprostatic extension, seminal vesicle invasion, perineural spread and vascular emboli, the low-grade histology, and the absence of PSMA-positive lesion in the prostate bed after prostatectomy and in the parotid gland area also supported the metachronous nature of the head and neck malignancy. The utilization of immunohistochemistry (IHC) in this context would not have been helpful, as SDC is reported to often duplicate the IHC profile of prostate adenocarcinoma regarding the expression of androgen receptor (AR), PSA, prostatic-specific acid phosphatase (PAP), alpha-methylacyl-CoA racemase (AMACR), and NKX3 in several studies [13,14]. The apocrine features were in favor of SDC as they are the norm in this entity and not previously described in prostate adenocarcinoma. Furthermore, PSA levels were <0.01 ng/dl and the Ga-68 PSMA PET/CT finding was negative both in the salivary gland tumor and in the metastatic foci.

Even a positive PSMA finding would not lead to the unequivocal diagnosis of metastatic prostate cancer in this setting, as several studies show variable PSMA uptake and PSMA IHC expression in salivary gland benign and malignant tumors (including SDC) [15,16]. Although there were no regional lymph nodes involved, the SDC demonstrated the classical pattern of distant spread with bone, lung, liver, and brain metastases, further aggravating the prognosis of the patient we presented.

## Conclusions

The presented case emphasizes the importance of the multidisciplinary management of oncological patients with more than one primary. The overlapping features of the discussed entities both on histological and immunohistochemical grounds can be a reason for substantial initial diagnostic uncertainty. This is shown in the current case, in which despite the initially relatively good prognosis of prostate adenocarcinoma without metastasis, following the surgical treatment of the parotid gland and the identification of ductal carcinoma within it, the prognosis significantly deteriorates with the discovery of multiple metastases and the progression of metastatic disease over time. This underscores the complexity of managing patients with multiple primary malignancies and the challenges in their diagnosis and treatment.

## References

[1] Filho OV, Rêgo TJ, Mendes FH (2022). Prognostic factors and overall survival in a 15-year followup of patients with malignant salivary gland tumors: a retrospective analysis of 193 patients. Braz J Otorhinolaryngol.

[2] Wierzbicka M, Kopeć T, Szyfter W, Kereiakes T, Bem G (2012). The presence of facial nerve weakness on diagnosis of a parotid gland malignant process. Eur Arch Otorhinolaryngol.

[3] Guntinas-Lichius O, Silver CE, Thielker J (2018). Management of the facial nerve in parotid cancer: preservation or resection and reconstruction. Eur Arch Otorhinolaryngol.

[4] Parikh AS, Khawaja A, Puram SV (2019). Outcomes and prognostic factors in parotid gland malignancies: a 10-year single center experience. Laryngoscope Investig Otolaryngol.

[5] Udager AM, Chiosea SI (2017). Salivary duct carcinoma: an update on morphologic mimics and diagnostic use of androgen receptor immunohistochemistry. Head Neck Pathol.

[6] Williams L, Thompson LD, Seethala RR (2015). Salivary duct carcinoma: the predominance of apocrine morphology, prevalence of histologic variants, and androgen receptor expression. Am J Surg Pathol.

[7] Nakaguro M, Tada Y, Faquin WC, Sadow PM, Wirth LJ, Nagao T (2020). Salivary duct carcinoma: updates in histology, cytology, molecular biology, and treatment. Cancer Cytopathol.

[8] Nachtsheim L, Mayer M, Meyer MF (2023). Incidence and clinical outcome of primary carcinomas of the major salivary glands: 10-year data from a population-based state cancer registry in Germany. J Cancer Res Clin Oncol.

[9] Nakaguro M, Sato Y, Tada Y (2020). Prognostic implication of histopathologic indicators in salivary duct carcinoma: proposal of a novel histologic risk stratification model. Am J Surg Pathol.

[10] Anwer AW, Faisal M, Adeel M (2018). Clinicopathological behavior and treatment-related outcome of rare salivary duct carcinoma: the Shaukat Khanum Memorial Cancer Hospital experience. Cureus.

[11] Simpson RH (2013). Salivary duct carcinoma: new developments--morphological variants including pure in situ high grade lesions; proposed molecular classification. Head Neck Pathol.

[12] Antunes HP, Parada B, Carvalho J (2018). Prognostic value of subclassification (pT2 stage) of pathologically organ-confined prostate cancer: Confirmation of the changes introduced in the 8th edition of the American Joint Committee on Cancer (AJCC) staging system. Arch Ital Urol Androl.

[13] Fan CY, Wang J, Barnes EL (2000). Expression of androgen receptor and prostatic specific markers in salivary duct carcinoma: an immunohistochemical analysis of 13 cases and review of the literature. Am J Surg Pathol.

[14] Takada N, Nishida H, Oyama Y (2020). Immunohistochemical reactivity of prostate-specific markers for salivary duct carcinoma. Pathobiology.

[15] Nishida H, Kondo Y, Kusaba T, Kadowaki H, Daa T (2022). Immunohistochemical reactivity of prostate-specific membrane antigen in salivary gland tumors. Head Neck Pathol.

[16] van Boxtel W, Lütje S, van Engen-van Grunsven IC (2020). (68)Ga-PSMA-HBED-CC PET/CT imaging for adenoid cystic carcinoma and salivary duct carcinoma: a phase 2 imaging study. Theranostics.

